# Ikaros as a downstream mediator of BCR blockade therapy in B-cell non-Hodgkin lymphoma

**DOI:** 10.18632/oncoscience.568

**Published:** 2022-11-28

**Authors:** Marcelo Lima Ribeiro, Emmanuel Normant, Gaël Roué

**Keywords:** B-NHL, Bruton’s tyrosine kinase, phosphoproteomics, Ikaros, combination therapies

B cell receptor (BCR) complex is essential for B cell development and function, being its downstream effectors involved in the regulation of several biological process such as immune response, cell growth, adhesion, differentiation, survival, cytoskeletal remodeling, and apoptosis [1]. Genetic events leading to BCR constitutive activation and consequent enhancement of tumor cell proliferation and survival, are frequently observed in B-cell non-Hodgkin lymphoma (B-NHL) patients [2]. In the last decade, numerous studies have highlighted that pharmacological targeting of Bruton’s tyrosine kinase (BTK), an apical component of the BCR axis, represents an excellent anti-tumor strategy in chronic lymphocytic leukemia (CLL) and in determined subtype of B-NHL including mantle cell lymphoma (MCL) and follicular lymphoma (FL). Most BTK inhibitors bind irreversibly to the ATP binding site of BTK at the Cys481 amino acid residue, forming a covalent bond that impairs the activity of the kinase [3]. The first-in-class irreversible inhibitor, ibrutinib, has demonstrated an exceptional clinical activity as a monotherapy in various subtypes of B-NHL with overall response rates ranging from 33% to 90.5% [4]. However, adverse events such as rash, diarrhea, bleedings, infections, and atrial fibrillation are common and usually associated with off-target effect of the drug on secondary kinases. Beside these limitations, different mechanisms including phospho-lipase C gamma 2 (PLCγ2) or non-canonical nuclear factor of kappa B (NFκB) activation, as well as cysteine-to-serine mutation at the inhibitor binding site (BTK^C481S^), can lead to ibrutinib resistance [5].

Among the different strategies developed to overcome ibrutinib off-target effects, the design of more selective agents recently led to the characterization of TG-1701, which BTK Kd (inactivation rate constant) is comparable to that of ibrutinib, but with a significant lower binding to EGFR, ITK, TXK, and JAK3 [6]. In our recent study, the antitumor activity of TG-1701 followed the same pattern than ibrutinib in a panel of B-NHL cell lines and mouse xenografts, including two NFκB- and BTK^C481S^-driven BTKi resistant models. More interestingly, a multi-omics characterization of primary samples from CLL patients receiving TG-1701 monotherapy, demonstrated for the first time that mass spectrometry-mediated determination of B-cell phosphoproteome can successfully distinguish between pre- and post-treatment samples, clustering these latter according to patient’s early clinical responses (early-responders vs late-responders), and that this approach represents a valuable tool for early monitoring of clinical response to BTK antagonists. This methodology also revealed that, in BTKi early-responders, drug efficacy was associated with neither its pharmacokinetic nor its pharmacodynamics properties, but rather with the phosphorylation status of the zinc-finger transcription factor, Ikaros. While both the nuclear localization and the transcriptional activity of this highly conserved factor with a key role in the control of lymphocyte specification and differentiation [7], were known to depend on BTK-mediated phosphorylation at Ser214/215 residues [8], we identified two previously undescribed residues (Ser442/445) which seemed to exert the same regulatory properties. Indeed, we observed that, upon TG-1701 treatment, the loss of p-Ser442/445 mark in patient’s peripheral blood mononuclear cells (PBMCs) was associated with a functional deregulation of Ikaros-dependent gene signatures. Similar observations were made in a panel of B-NHL cell lines, including a set of CRISPR-Cas9-edited models overexpressing BTK^C481S^ or depleted in either BTK (BTK^KO^) or Ikaros (IKZF1^KO^). In these latter, we highlighted that TG-1701-mediated BTK blockade directly impairs Ikaros nuclear translocation, leading to the upregulation of the IKZF1-repressed gene, *YES1*, and the downregulation of the IKZF1-enhanced gene, *MYC*, two genes which were further shown to represent bona-fide markers of the superior selectivity of TG-1701 against BTK (Figure 1).

**Figure 1:**
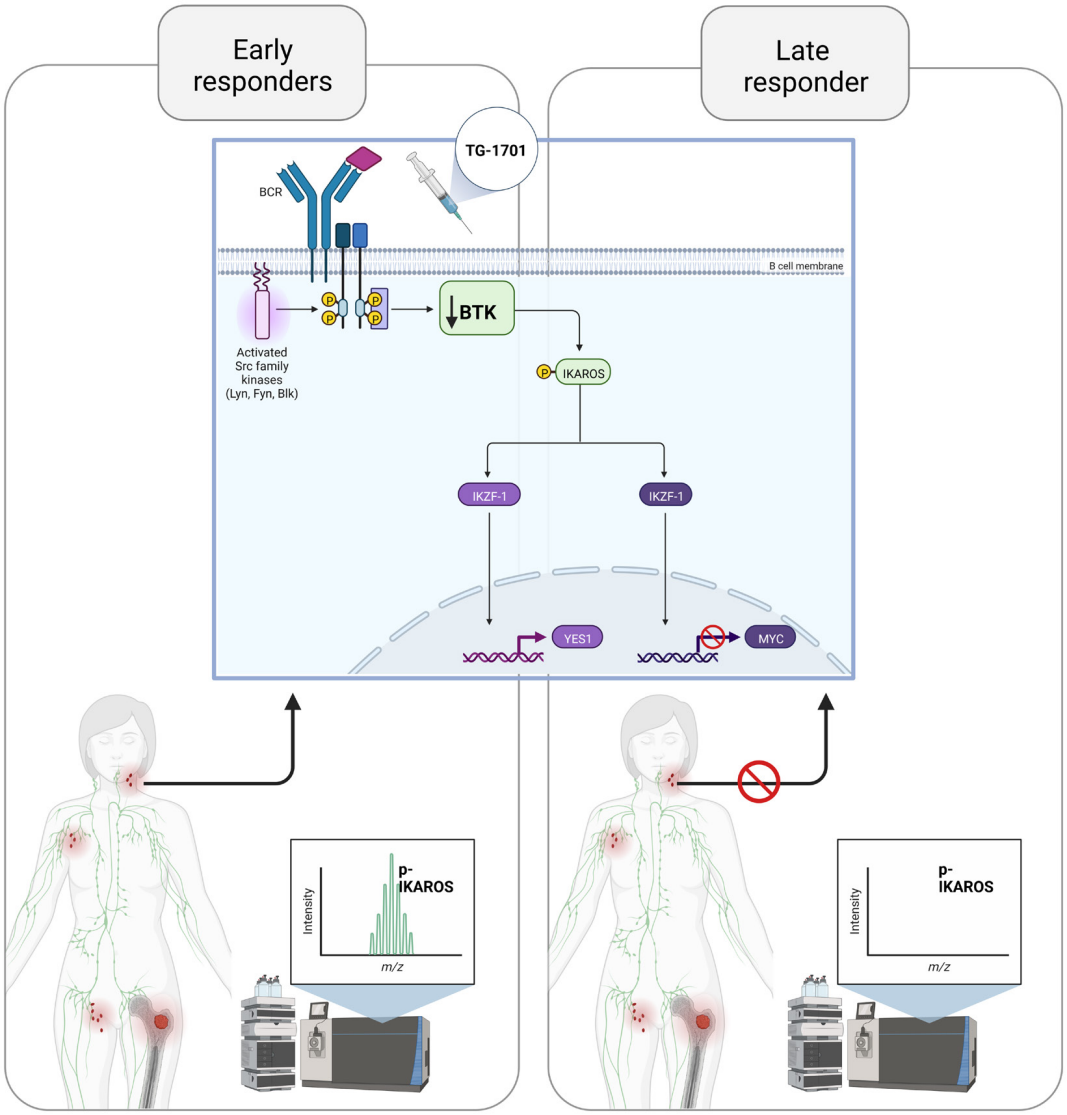
Early clinical response to the selective BTK inhibitor TG-1701 relies on the disruption of Ikaros transcriptional program.

Altogether, our data validated phosphoproteomic as a valuable tool for the monitoring of early clinical response to BTK inhibitors, for the determination of drug mechanism of action, and for the discovery of clinically-relevant biomarkers.

## References

[1] Kraus M, et al. Cell. 2004; 117:787–800. 10.1016/j.cell.2004.05.014. 15186779

[2] Küppers R, et al. Nat Rev Cancer. 2005; 5:251–62. 10.1038/nrc1589. 15803153

[3] Honigberg LA, et al. Proc Natl Acad Sci U S A. 2010; 107:13075–80. 10.1073/pnas.1004594107. 20615965 PMC2919935

[4] Xue C, et al. Cancer Cell Int. 2020; 20:467. 10.1186/s12935-020-01518-y. 33005100 PMC7523373

[5] Profitós-Pelejà N, et al. Cancers (Basel). 2022; 14:860. 10.3390/cancers14040860. 36428695 PMC9688202

[6] Ribeiro ML, et al. Clin Cancer Res. 2021; 27:6591–601. 10.1158/1078-0432.CCR-21-1067. 34551904 PMC9401565

[7] Schjerven H, et al. Nat Immunol. 2013; 14:1073–83. 10.1038/ni.2707. 24013668 PMC3800053

[8] Ma H, et al. PLoS One. 2013; 8:e71302. 10.1371/journal.pone.0071302. 23977012 PMC3747153

